# Apoptosis is not the major death mechanism induced by celecoxib on rheumatoid arthritis synovial fibroblasts

**DOI:** 10.1186/ar2342

**Published:** 2007-12-12

**Authors:** Rachel Audo, Véronique Deschamps, Michael Hahne, Bernard Combe, Jacques Morel

**Affiliations:** 1 Institut de Génétique Moléculaire de Montpellier, 1919 route de Mende, CNRS UMR5535, Montpellier, France; 2 Service d'immuno-rhumatologie et Université Montpellier 1, 371 Ae du doyen Gaston Giraud, Montpellier, France

## Abstract

Synovial hyperplasia in rheumatoid arthritis (RA) has been associated with apoptosis deficiency of RA fibroblast-like synoviocytes (FLSs). Celecoxib is a non-steroidal anti-inflammatory drug that has been demonstrated to induce apoptosis in some cellular systems. We have therefore examined the dose- and time-dependent effects of celecoxib on RA FLS viability. Treatment of RA FLSs with celecoxib for 24 hours reduced their viability in a dose-dependent manner. Analysis of celecoxib-treated RA FLSs for their content of apoptotic and necrotic cells by Annexin V staining and TO-PRO-3 uptake displayed only few apoptotic cells. Caspase 3, a key mediator of apoptosis, was not activated in celecoxib-treated RA FLSs, and the presence of specific caspase 3 or pan-caspase inhibitors did not affect celecoxib-induced cell death. Moreover, we could not detect other signs of apoptosis, such as cleavage of poly(ADP-ribose) polymerase, caspase 8 or 9, or DNA fragmentation. We therefore conclude that apoptosis is not the major death pathway in celecoxib-treated RA FLSs.

## Introduction

Cyclooxygenases (COXs) are key enzymes in the conversion of arachidonic acid to prostanoids, which mediate mitogenesis, apoptosis, angiogenesis, blood flow, secondary injury (lipid peroxidation and oxidative stress), and inflammation [1]. The COX-1 isoform is constitutively expressed under physiological conditions, whereas expression of the COX-2 isoform is inducible under pathophysiological, mainly inflammatory, conditions [2]. Consequently, the current pharmacological strategy is to selectively inhibit COX-2 and thereby avoid unfavorable effects of combined COX-1 and COX-2 blocking [1,2].

Rheumatoid arthritis (RA) is an autoimmune disease characterized by chronic inflammation of joints, leading to a progressive and irreversible joint destruction [3,4]. The aggressive front of synovial tissue, called pannus, invades and destroys the local articular structure [3,4]. The pannus is characterized by a synovial hyperplasia that is mainly composed of fibroblast-like synoviocytes (FLSs) combined with a massive infiltration of lymphocytes and macrophages [3,4]. Increased proliferation and insufficient apoptosis might contribute to the expansion of RA FLSs, and several reports suggest inducing apoptosis of RA FLSs as a therapeutic approach [3,4].

Celecoxib (4-[5-(4-methylphenyl)-3-(trifluoromethyl)-1H-pyrazol-1-yl] benzenesulfonamide) is an anti-inflammatory drug that specifically inhibits the COX-2. Celecoxib has been described as a pro-apoptotic factor in several human carcinoma cells [5-7]. In addition, it has been reported that high doses of celecoxib have a pro-apoptotic effect on RA FLSs [8]. Here, we report that the cell death induced by high doses of celecoxib on RA FLSs is rapid without displaying characteristics of apoptosis.

## Materials and methods

### Reagents

Celecoxib and valdecoxib were generously provided by Pfizer Inc (New York, NY, USA) and dissolved in dimethyl sulfoxide (DMSO) at 100 mM. Indomethacin (Sigma-Aldrich, St Quentin Fallavier, France) was dissolved in ethanol at a final concentration of 100 mM. Pan-caspase inhibitor (z-VAD-fmk [benzyloxycarbonyl-Val-Ala-Asp (OMe) fluoromethylketone]), caspase 3 inhibitor (z-DEVD-fmk [benzyloxycarbonyl-Asp(OMe)-Glu(OMe)-Val-Asp(OMe)-FMK inhibitor]), and the caspase control inhibitor z-FA-fmk (benzyloxycarbonyl-phenyl-alanyl-fluoromethylketone) specific for cathepsins B and L (R&D Systems, Lille, France) were dissolved at 20 mM in DMSO. Annexin V was purchased from Roche Diagnostic (Meylan, France) and TO-PRO-3 from Invitrogen Corporation (Cergy Pontoise, France). Anti-caspase antibodies were obtained from Cell Signaling Technology (St Quentin Yveline, France), poly(ADP-ribose) polymerase (PARP) antibody from BD Pharmingen (BD Biosciences, Le-Pont-de-Claix, France), and peroxidase-conjugated secondary antibodies were purchased from Sigma-Aldrich.

### Preparation of fibroblast-like synoviocytes of patients with rheumatoid arthritis

Fibroblasts were isolated from synovium obtained from patients who met the American College of Rheumatology criteria for RA (revised 1987) and who had undergone surgery for synovectomy or total joint replacement surgery [9]. Fresh synovial tissues were minced and digested in a solution of dispase (Gibco, now part of Invitrogen Corporation) and collagenase (Sigma-Aldrich) and DNase (Calbiochem, now part of EMD Biosciences, Inc., San Diego, CA, USA). Synovial fibroblasts were cultured in RPMI 1640 supplemented with 10% fetal calf serum (FCS). Cells were used at passages 4 to 10, when they constitute a homogeneous population of fibroblasts, free of detectable T cells or macrophages. Upon reaching confluence, the cells were passaged by brief trypsinization. For experimentation, the content of FCS in the media was progressively decreased from 10% to 1% with final starvation for 12 hours in RPMI 1640 media containing 1% FCS, as described previously [10].

### Analysis of cell viability and apoptosis

Cell viability was measured by taking metabolic activity as a readout using the Celltiter 96 AQueous cell proliferation (MTS) assay (Promega Corporation, Charbonnières, France) after 24 hours of cell culturing according to the manufacturer's instructions. Apoptotic RA FLSs were identified by resuspending 1 × 10^5 ^cells in 100 μL of Annexin V Binding buffer containing 5 μL of Annexin V-fluorescein isothiocyanate (10 μg/mL; R&D Systems) for 15 minutes at room temperature. Upon addition of TO-PRO-3 (1:2,000), cells were analyzed by flow cytometry (FACSCalibur; BD Biosciences) [11].

### Cell proliferation assay

Proliferation was evaluated measuring DNA synthesis by incorporation of tritiated [^3^H]thymidine. FLSs were seeded in 96-well flat-bottom culture plates at a density of 1 × 10^4 ^cells per well. Cells were cultured in RPMI 1640 with decreasing concentrations of FCS (10% and 5%) and then synchronized for 24 hours with RPMI 1640 and 1% FCS. FLSs were stimulated for 72 hours. Every condition was tested in quadruplicate. [^3^H]thymidine (1 μCi/well) was added 24 hours before the end of the assay. FLSs were lysed using a round of freeze-thaw cycle and then transferred onto a membrane filter using Harvester 96 (Tomtec, Hamdem, CT, USA). [^3^H]thymidine incorporated into DNA was quantified using a scintillation counter 1450 MicroBeta Trilux (Wallac, now part of PerkinElmer Life and Analytical Sciences, Inc., Waltham, MA, USA').

### Western blotting analysis

Synovial cells were seeded in six-well plates at 2 × 10^5 ^cells per well or in 6-cm dishes at 4 × 10^5 ^cells. After serum starvation, RA FLSs were treated as indicated. Both detached and adherents cells were collected, washed twice with cold phosphate-buffered saline (PBS), and treated with lysis buffer as described previously [10]. Total cell extracts were resolved by SDS-PAGE and proceeded for immunoblot analysis as described previously [12]. Primary antibodies were diluted according to the manufacturer's instructions (1:1,000 dilutions for anti-caspase and anti-PARP antibodies and 1:5,000 dilution for anti-β-actin antibody), and nitrocellulose membranes were incubated overnight at 4°C with the primary antibodies (or for 1 hour at room temperature for β-actin). Equal loading was confirmed by β-actin expression.

### Ac-DEVD-AMC (caspase 3/7 fluorogenic substrate) protease assay

Synovial fibroblasts were seeded in six-well plates at 2 × 10^5 ^cells per well. After serum starvation, RA FLSs were treated with either celcoxib or tumor necrosis factor-related apoptosis-inducing ligand (TRAIL), and caspase 3 activation was measured using the Ac-DEVD-AMC protease assay according to the manufacturer's instructions (BD Biosciences).

### DNA fragmentation

FLSs were seeded in 96-well flat-bottom culture plates at 1 × 10^4 ^cells per well, cultured with decreasing concentrations of FCS (10%, 5%, and 1%) as described above, and incubated for 12 hours with either celecoxib or TRAIL. Cells were collected, washed with PBS, and processed for quantification of DNA fragments using an enzyme-linked immunosorbent assay according to the manufacturer's instructions (Cell Death Detection ELISA^PLUS^; Roche Diagnostic).

## Results

### Celecoxib decreases cell activity and proliferation of rheumatoid arthritis fibroblast-like synoviocytes

**Figure 1:**
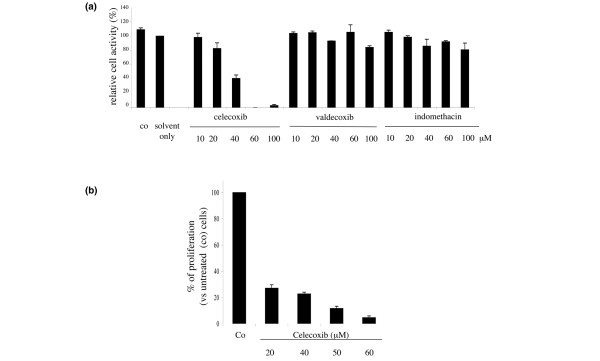
Celecoxib reduces viability and proliferation of synovial fibroblasts extracted from patients with rheumatoid arthritis (RA). **(a) **Fibroblast-like synoviocytes (FLSs) of patients with RA were cultured for 24 hours in the presence of the cyclooxygenase inhibitors celecoxib, valdecoxib, or indomethacin at the indicated concentrations. Metabolic activity was determined by means of the MTS assay. Untreated cells (Co) and cells treated only with solvent served as controls. The graph presents relative cell viability toward cells treated only with solvent as the mean ± standard error of the mean (SEM) of three individual experiments. **(b) **Celecoxib strongly inhibits RA FLS proliferation in a dose-dependent manner. RA FLSs were stimulated for 24 hours with the indicated concentrations of celecoxib, and proliferation was assessed using [^3^H]thymidine incorporation. The graph presents relative cell viability toward cells treated only with solvent as the mean ± SEM of three individual experiments.

We first analyzed the effect of celecoxib on metabolic activity of RA FLSs. For this, RA FLSs were treated for 24 hours with different concentrations of celecoxib and subsequently analyzed for cell activity using MTS assay. Cell activity of RA FLSs was clearly reduced by the addition of 40 μM celecoxib and completely abrogated in the presence of 60 μM (Figure 1a). RA FLS viability was only modestly affected by lower concentrations of celecoxib. Indomethacin, an inhibitor for both COX-1 and COX-2, and valdecoxib, another specific COX-2 inhibitor, had no effect on RA FLS activity, demonstrating that the observed effect is specific for celecoxib.

The decreased viability of celecoxib-treated RA FLSs was mirrored by their decreased proliferation capacity as measured by thymidine incorporation. Celecoxib inhibited RA FLS proliferation in a dose-dependent manner at all of the concentrations tested (20, 40, 50, and 60 μM), and only marginal thymidine incorporation was detectable in the presence of 60 μM celecoxib (Figure 1b).

### Celecoxib-treated rheumatoid arthritis fibroblast-like synoviocytes bypass the state of early apoptosis

**Figure 2:**
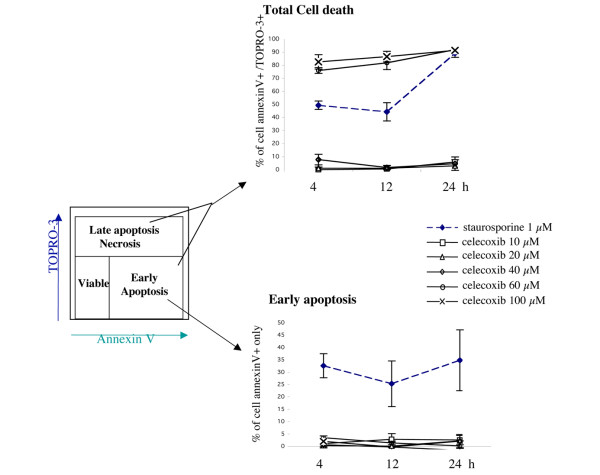
Celecoxib induces cell death in rheumatoid arthritis fibroblast-like synoviocytes. Cells were treated at indicated concentrations and times with either celecoxib or staurosporine. Apoptosis was evaluated by fluorescence-activated cell sorting analysis using Annexin V binding and TO-PRO-3 uptake. Values are expressed as the percentage of total cell death (upper panel) or apoptosis (lower panel) and are the mean ± standard error of the mean of three individual experiments.

We next analyzed celecoxib-treated RA FLSs for their content of apoptotic and necrotic cells by Annexin V staining and TO-PRO-3 uptake. This technique allows investigators to distinguish early apoptotic cells (Annexin^+^/TO-PRO-3^-^) from late apoptotic/necrotic cells (Annexin^+^/TO-PRO-3^+^) [11] (Figure 2).

**Figure 3:**
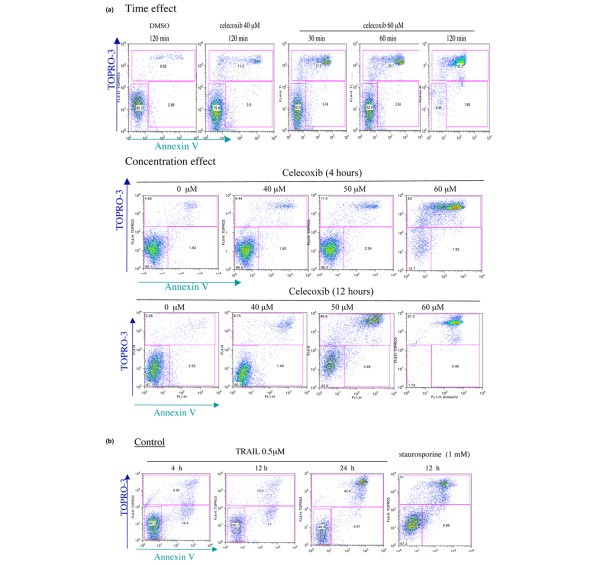
Characterization of celecoxib-induced cell death in rheumatoid arthritis fibroblast-like synoviocytes. Cells were treated at indicated concentrations and times with **(a) **celecoxib or **(b) **staurosporine or tumor necrosis factor-related apoptosis-inducing ligand. Apoptosis was evaluated by fluorescence-activated cell sorting analysis using Annexin V binding and TO-PRO-3 uptake. Representative data of three different experiments are shown. DMSO, dimethyl sulfoxide.

RA FLSs were treated with different concentrations (10 to 100 μM) of celecoxib, and cell death was observed only at celecoxib concentrations of at least 60 μM (Figure 2). Valdecoxib, used at the same concentration, induced no cell death (data not shown). Twenty-four hours of treatment of RA FLSs with 60 μM celecoxib induced death in nearly all cells, which displayed the characteristics of late apoptotic/necrosis (that is, were Annexin^+ ^and TO-PRO-3^+^). A similar pattern was observed after 4 hours of treatment with celecoxib, when no early apoptotic cells (Annexin V^+ ^and TO-PRO-3^-^) were detectable (Figure 2). Moreover, when RA FLSs were treated for shorter time points (30 minutes and 1 and 2 hours) with 60 μM celecoxib, no early apoptotic cells were detectable, whereas cells were already detectable in the Annexin^+ ^and TO-PRO-3^+ ^gate (Figure 3a). Two hours of treatment of RA FLSs with 60 μM celecoxib was sufficient to induce cell death in more than 90% of the cells, whereas only 11% dead cells were detectable upon treatment with 40 μM celecoxib. Intermediate concentrations between 40 and 60 μM demonstrated that celecoxib induces cell death in a dose-dependent manner and this cell death is also characterized by an immediate shift into the necrosis/late apoptosis gate (Figure 3a). Although the percentage of dead cells detectable on celecoxib-treated cells varied between the FLSs of different patients, it never reached those differences detected by measuring cell viability (Figure 1). This is most likely due to the fact that the MTS assay used for measuring viability quantifies the metabolic activity of cells and that metabolically inactive cells are not necessarily dead cells.

**Figure 4:**
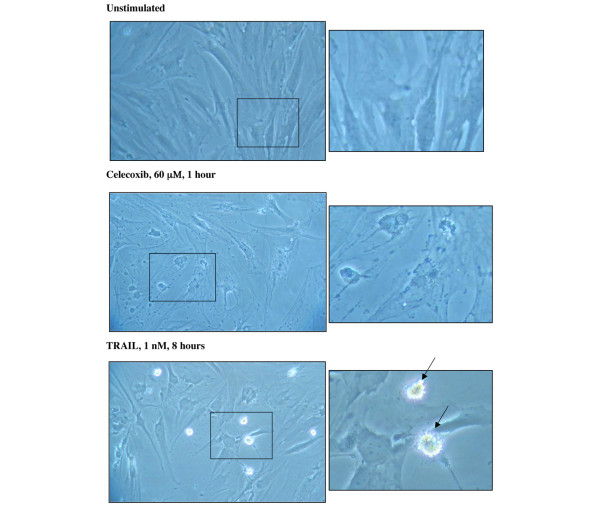
Comparison of morphological changes of rheumatoid arthritis fibroblast-like synoviocytes (RA FLSs) treated with either celecoxib or tumor necrosis factor-related apoptosis-inducing ligand (TRAIL) by light microscopy (magnification × 300). Untreated RA FLSs (upper panel) and cells treated for either 1 hour with 60 μm celecoxib (middle panel) or 8 hours with 1 nM TRAIL (lower panel) are shown. Apoptotic cells are indicated by arrows.

To validate our approach for the detection of apoptotic cells, we treated RA FLSs with staurosporine and TRAIL (also called APO-2L). Staurosporin is a non-selective protein kinase inhibitor that is known to induce apoptosis in several cell types [13], whereas TRAIL is a member of the tumor necrosis factor family which induces apoptosis in a wide variety of tumor cells as well as RA FLSs [10]. Indeed, early apoptotic cells were observed in cells that were treated for 4 hours with either staurosporine or TRAIL (Figure 3b). TRAIL-treated RA FLSs displayed morphological changes characteristic for apoptosis, including cell shrinkage and membrane blebbing, that were not detectable on celecoxib-treated RA FLSs. Celecoxib initially induced a compression of the cells which was followed by cellular swelling associated with the formation of dendritic-like structures (Figure 4).

### No caspase activity is detectable in celecoxib-treated rheumatoid arthritis fibroblast-like synoviocytes

One mechanism that is consistently implicated in apoptosis is the activation of a cascade of cytosolic proteases called caspases. Caspases are synthesized as inactive proenzymes that are processed by proteolytic cleavage to form an active enzyme. A member of this family, caspase 3 (CPP32, apopain, and YAMA), plays a central role in the execution of apoptosis in mammalian cells, and activation of caspase 3 is therefore a hallmark of apoptotic cells [14].

**Figure 5:**
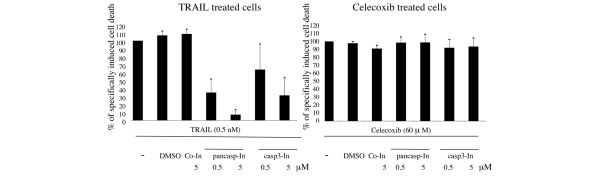
Celecoxib-induced cell death in rheumatoid arthritis fibroblast-like synoviocytes is caspase-independent. Effect of caspase inhibition on celecoxib-induced cell death. Cells were pre-treated with caspase inhibitors (pancasp-In: pan-caspase inhibitor z-VAD-fmk; casp3-In: caspase 3 inhibitor z-DEVD-fmk) or control inhibitor z-FA-fmk (co-In: control inhibitor) for 1 hour and subsequently cultured in the presence of either 60 μM celecoxib or 0.5 nM tumor necrosis factor-related apoptosis-inducing ligand (TRAIL) for an additional 24 hours. Cells pre-treated only with solvent (dimethyl sulfoxide [DMSO]) served as controls. Cell death was determined using Annexin V binding and TO-PRO-3 uptake and expressed as relative cell death. (For this, cell death induced by TRAIL or celecoxib plus inhibitor was first subtracted by cell death of cells treated with inhibitor alone and then expressed as percentage versus cell death induced by TRAIL or celecoxib alone.) Data from three patients were averaged and are shown as the mean ± standard error of the mean.

**Figure 6:**
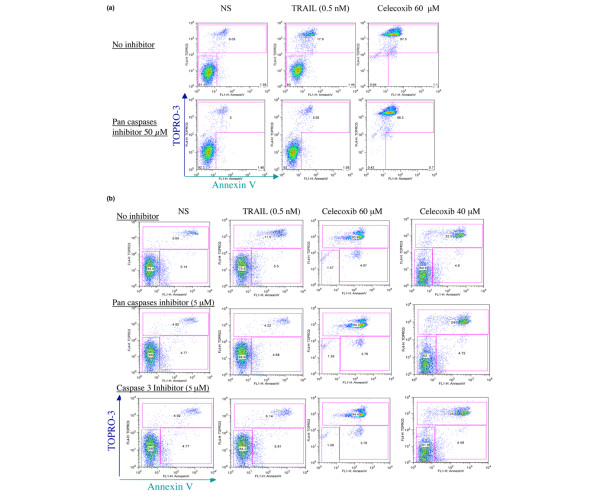
Celecoxib-induced cell death in rheumatoid arthritis fibroblast-like synoviocytes is caspase-independent. Effect of caspase inhibition on celecoxib-induced cell death using a higher concentration of caspase inhibitor **(a) **and a lower concentration of celecoxib (40 μM) **(b)**. In these conditions, inhibition of cell death could not be observed. Representative data of three different experiments are shown. NS, unstimulated cells; TRAIL, tumor necrosis factor-related apoptosis-inducing ligand.

To assess the contribution of caspases in celecoxib-mediated cytotoxicity, RA FLSs were treated with the pan-caspase inhibitor z-VAD-fmk or with z-DEVD-fmk, a specific inhibitor of caspase 3, and subsequently with celecoxib (60 μM). A 5-μM concentration of neither a pan-caspase inhibitor nor the specific caspase 3 inhibitor could protect RA FLSs against celecoxib-induced cell death, whereas cell death induced by TRAIL was significantly reduced by a 5-μM concentration of either pan-caspase inhibitor z-VAD-fmk or caspase 3 inhibitor (Figure 5). Also, higher concentrations (50 μM) of the pan-caspase inhibitor z-VAD-fmk had no inhibitory effect on celecoxib-induced cell death (Figure 6a). Caspase inhibitors also did not affect cell death of RA FLSs treated with lower concentrations (40 μM) of celecoxib (Figure 6b).

**Figure 7:**
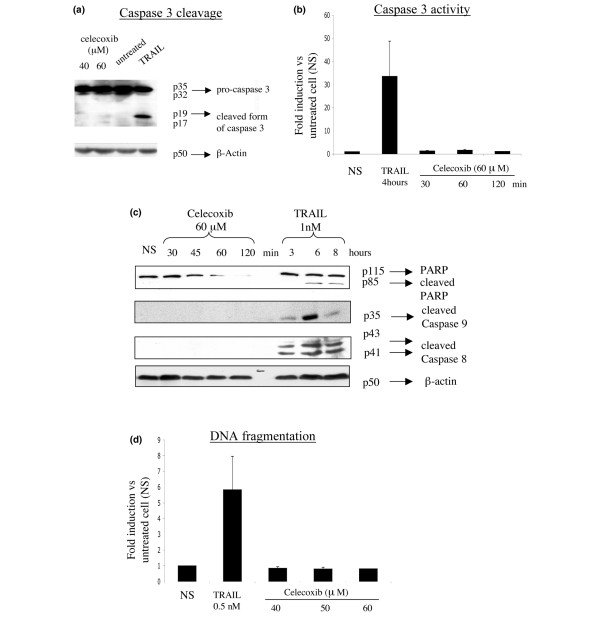
Celecoxib does not induce caspase activation in rheumatoid arthritis fibroblast-like synoviocytes (RA FLSs). **(a) **FLSs were stimulated for 2 hours with celecoxib at indicated concentrations or for 4 hours with tumor necrosis factor-related apoptosis-inducing ligand (TRAIL) (0.5 nM) as positive control. Cell lysates were analyzed by immunoblot for caspase 3 expression. The same blot was stripped and reprobed with a mouse anti-human β-actin antibody to confirm equal loading. One representative immunoblot is shown. **(b) **RA FLSs were stimulated for indicated time points with 60 μM celecoxib or with TRAIL (0.5 nM) as positive control, and caspase 3 activity was measured using Ac-DEVD-AMC protease assay. Caspase 3 activity is expressed as fold increase to unstimulated cells (NS) and is represented as the mean ± standard error of the mean (SEM) of different experiments using RA FLSs from three different patients. **(c) **RA FLSs were stimulated for indicated time points with celecoxib at indicated concentrations or with TRAIL (0.5 nM) as positive control. Cell lysates were analyzed by immunoblot for poly(ADP-ribose) polymerase (PARP) and caspase 8 and 9 expression. One representative immunoblot is shown. **(d) **RA FLSs were stimulated for 12 hours with celecoxib at indicated concentrations or with TRAIL (0.5 nM) as positive control, and DNA fragmentation was measured using the Cell Death Detection ELISA^PLUS ^kit. The enrichment of mono- and oligonucleosomes released into the cytoplasm is calculated as the ratio of the absorbance of the sample cells to the absorbance of control cells and is shown as the mean ± SEM from three experiments performed in duplicate.

Cleavage of caspases is an indicator for their activation. We therefore analyzed whether the cleaved forms of caspases 3, 8, and 9 were detectable in celecoxib-treated RA FLSs. Cell death happened faster in RA FLSs treated with celecoxib than in those treated with TRAIL. For this reason, we compared RA FLSs treated for 2 hours with celecoxib and cells treated for at least 3 hours with TRAIL. The cleaved forms of caspase 3, 8, and 9 were not detectable in cell lysates of RA FLSs treated with celecoxib but were detectable in those treated with TRAIL (Figure 7a,c). Finally, we analyzed the caspase 3 activity using an Ac-DEVD-AMC protease assay in celecoxib- and TRAIL-treated cells but detected caspase 3 activity only in TRAIL-treated cells (Figure 7b).

Active caspase 3 proteolytically cleaves and activates, among other targets, PARP involved in DNA repair and DFF40/CAD DNase, the executor of nuclear DNA fragmentation. Apoptotic cells are characterized by cleavage of the native 116-kDa form of PARP into 85-kDa and 25-kDa forms. Concurring with the observed absence of the active form of caspase 3, PARP cleavage was not observed in celecoxib-treated RA FLSs but was observed in those treated with TRAIL (Figure 7c). Moreover, we could not detect DNA fragmentation in RA FLSs treated for either 12 or 24 hours with celecoxib concentrations of 40, 50, or 60 μM, but we could in TRAIL-treated cells (Figure 7d). Taken together, our results suggest that the cell death pathway induced by celecoxib on RA FLSs occurs in a caspase-independent manner.

## Discussion

Apoptosis is a form of cell death in which a programmed sequence of events leads to the elimination of cells without releasing harmful substances into the surrounding area. Apoptosis plays a crucial role in controlling cell numbers by eliminating old cells, unnecessary cells, and unhealthy cells. Deregulation of apoptosis thus can lead to the survival and hyperproliferation of unwanted cells such as FLSs in RA. Therefore, one strategy for treatment is the design of drugs that can restore the normal apoptotic pathways in hyperproliferative cells.

The anti-inflammatory drug celecoxib, an inhibitor of COX-2, was reported by Kusunoki and colleagues [8] to be pro-apoptotic on RA FLSs. In that study, the viability of synovial cells was reduced by celecoxib in a dose-dependent manner similar to our observation. Kusunoki and colleagues observed that celecoxib strongly reduced cell viability of RA FLSs when used at concentrations of at least 30 μM. The authors concluded that celecoxib induces apoptosis in RA FLSs as they observed a strong DNA fragmentation in RA FLSs treated with 30 μM celecoxib.

We compared the characteristics of celecoxib-induced cell death in RA FLSs with those induced by the established pro-apoptotic factor TRAIL by Annexin V staining/TO-PRO-3 uptake. We have previously reported that 4 hours of co-culturing with TRAIL resulted in apoptosis of approximately 30% of RA FLSs as determined by Annexin V staining/TO-PRO-3 uptake. This technique allows investigators to distinguish early apoptotic cells (Annexin^+^/TO-PRO-3^-^) from late apoptotic/necrotic cells (Annexin^+^/TO-PRO-3^+^) [11]. Annexin V is a Ca^2+^-dependent phospholipid-binding protein with high affinity for phospatidylserine and can be used as a sensitive probe for the early phase of apoptosis that is characterized by phospatidylserine exposure on the cell membrane. Because of increased permeability, Annexin V binding can also occur during cell necrosis, and uptake of DNA stain TO-PRO-3 is taken as a parameter to distinguish necrotic, and thus permeable, cells [11].

Whereas we observed pre-apoptotic cell death in RA FLSs upon 4 hours of TRAIL treatment, pre-apoptotic cells were hardly detectable in celecoxib-treated cells. We exposed RA FLSs to different concentrations of celecoxib for various incubation times, but under none of the tested conditions were pre-apoptotic (that is, Annexin V^+ ^and TO-PRO-3^-^) cells detectable. Cell death was observed only at concentrations above 40 μM celecoxib (Figure 2), although we detected a decrease in the metabolic activity of FLSs treated with lower concentrations of celecoxib, which is in agreement with Kusunoki and colleagues [8]. We also confirm that celecoxib strongly inhibited RA FLS proliferation, as shown in their study [8].

It has been suggested that cell death should be classified as apoptosis only if execution of cell death is dependent on caspase activity [14]. We therefore tested whether caspase 3 is activated in celecoxib-treated cells as caspase 3 is a key executor of apoptosis. Whereas the cleaved (thus active) form of caspase 3 was detectable in cell lysates of TRAIL-treated RA FLSs, only the proform of caspase 3 was visible in celecoxib-treated cells. Concurring with this observation, neither a pan-caspase inhibitor nor a specific caspase 3 inhibitor could protect RA FLSs against celecoxib-induced cell death. Similar observations were made with caspase 8 and caspase 9 inhibitors (data not shown). We therefore conclude that celecoxib-induced cell death in RA FLSs is independent of caspases.

These results are in contrast to those of Kusunoki and colleagues [8], who observed DNA fragmentation in RA FLSs treated for 24 hours with celecoxib and an inhibition of this DNA fragmentation by the addition of caspase 3 inhibitors. A possible explanation for these differences might be the different cell systems used. Kusunoki and colleagues used the first two passages of fibroblasts prepared from synovial tissue for their experimentation. We, however, employed RA FLSs between passages 4 and 10 in order to exclude a contamination of other cell types because approximately 30% of the synovium is composed of macrophage-like synoviocytes.

Moreover, evidence is accumulating that taking DNA fragmentation as the readout of caspase inhibition is not sufficient to conclude the contribution of caspases to cell death [15]. It is therefore possible that the caspase dependency of DNA fragmentation described by Kusunoki and colleagues is a secondary event during celecoxib-induced cell death, which could also explain their observed dose-independent effect of the caspase inhibitors. To conclude an apoptotic cell death, other parameters of cell death such as mitochondrial dysfunction, phosphatidylserine exposure, and plasma membrane permeabilization have to be considered [15]. Nevertheless, DNA fragmentation can also be observed in non-apoptotic cell death pathways, as for example during necrosis [16].

## Conclusion

We conclude from our results that apoptosis is not the major death pathway in RA FLSs induced by high concentrations of celecoxib. This observation is in line with that of a previous report, in which celecoxib was described as being able to induce non-apoptotic cell death in cardiac myocytes [17]. Administration of antioxidants, such as dithiothreitol, *N*-acteyl-cysteine, or calcium-blocking reagents, did not alter celecoxib-induced cell death, excluding an implication of oxidative stress and intracellular calcium signals (data not shown). The morphological changes together with the rapid membrane permeabilization of RA FLSs during celecoxib treatment suggest rather a necrotic-like cell death. Necrosis, however, leads to the release of inflammatory cellular contents which is unfavorable in the treatment of RA. The proposed local administration of higher celecoxib concentrations in the synovial space of the joint of arthritis patients [8] must therefore be reconsidered.

## Abbreviations

COX = cyclooxygenase; DMSO = dimethyl sulfoxide; FCS = fetal calf serum; FLS = fibroblast-like synoviocyte; PARP = poly(ADP-ribose) polymerase; PBS = phosphate-buffered saline; RA = rheumatoid arthritis; TRAIL = tumor necrosis factor-related apoptosis-inducing ligand; z-DEVD-fmk = benzyloxycarbonyl-Asp(OMe)-Glu(OMe)-Val-Asp(OMe)-FMK inhibitor; z-VAD-fmk = benzyloxycarbonyl-Val-Ala-Asp (OMe) fluoromethylketone.

## Competing interests

The authors declare that they have no competing interests.

## Authors' contributions

RA performed the experimental work and the analysis of the data and participated in the writing of the manuscript. VD performed several experiments. MH participated in the analysis of the study and in the writing of the manuscript. BC and JM participated in the design of the study and in the writing of the manuscript. All authors read and approved the manuscript.
